# Supplementary material for: Ten Thousand-Fold Higher than Acceptable Bacterial Loads Detected in Kenyan Hospital Environments: Targeted Approaches to Reduce Contamination Levels
Source: Int J Environ Res Public Health. 2021 Jun 25;18(13):6810. doi: 10.3390/ijerph18136810 (PMC8297338; doi:10.3390/ijerph18136810)
Supplement: Supplementary file 1 [file ijerph-18-06810-s001.zip › ijerph-1254105-supplementary.pdf]

Supplementary Table S1: Number and types of high touch surfaces or items sampled in the study (N =559).

Patient and clinical areas	No. of sampled items	Equipment	No. of sampled items	Bathroom Areas	No. of sampled items
Patient beds	92	Intravenous pole handles and tubing	68	Bathroom sinks	12
Bed rails	79	Baby weighing scales	13	Toilet handrails	4
Room inner doorknobs	45	Computer keyboards	6	Toilet flush handles	4
Room sinks	44	Telephone dial pad	8	Toilet flush buttons	2
Newborn incubators	25	Oxygen concentrator dial pads	4	Saline bath tub	2
Tray table tops	23	Computer mouse	4		
Room light switch plates	21	Blood pressure cuffs	4		
Bedside table tops	13	Thermometers	2		
Patient chairs - arms	12	Baby height meters	2		
Baby cots	10	Adult weighing scale pole handles	2		
Clinicians laboratory coats	8	Manual vacuum aspiration patient beds	1		
Clinicians desks	7	IV tubing	2		
Patient record holders	4	Fridge door handles	2		
Operation gowns – midsections & cuffs	4	Equipment Control panels	2		
Cupboard handles	4	Clinician mobile phones	2		
Trolley handles	2	Call system dial pad	2		
Stretcher	2	Suction tubes	2		
Patient examination beds	2	Stethoscope	2		
Operation table handles	2	Oxygen tubes	2		
Operation tables	2				
Breast feeding chairs	2				
Bed screeners	2				
**Total**	**405/559**		**130/559**		**24/559**
